# Dual Therapies Containing an Antibiotic Plus a Proton Pump Inhibitor or Vonoprazan for *Helicobacter pylori* Infection: A Systematic Review

**DOI:** 10.3390/microorganisms13040715

**Published:** 2025-03-21

**Authors:** Chih-An Shih, Deng-Chyang Wu, Chang-Bih Shie, Ping-I Hsu

**Affiliations:** 1 Division of Gastroenterology and Hepatology, Department of Internal Medicine, Antai Medical Care Corporation, Antai Tian-Sheng Memorial Hospital, Pingtung County 928004, Taiwan; dreric921@gmail.com; 2 Department of Nursing, Meiho University, Pingtung County 912009, Taiwan; 3 Division of Gastroenterology, Department of Internal Medicine, Kaohsiung Medical University Hospital, Kaohsiung City 807377, Taiwan; dechwu@yahoo.com; 4 Department of Medicine, College of Medicine, Kaohsiung Medical University, Kaohsiung City 807378, Taiwan; 5 Division of Gastroenterology, Department of Internal Medicine, An Nan Hospital, China Medical University, Tainan City 709204, Taiwan

**Keywords:** *Helicobacter pylori*, high-dose dual therapy, triple therapy, quadruple therapy, potassium competitive acid blocker

## Abstract

Due to the increasing prevalence of antimicrobial resistance, the efficacy of standard triple therapy for *Helicobacter pylori* (*H. pylori*) infection has declined, with eradication rates now falling below 80% in most countries. Although bismuth quadruple therapy and concomitant therapy are advised in regions with high clarithromycin resistance, these treatments commonly cause frequent adverse events and require the use of two or three antibiotics. This review article evaluates the effectiveness of 14-day mono-antibiotic therapies for *H. pylori* infection through randomized controlled trials conducted from 1 October 2014 to 1 October 2024. The pooled eradication rates for 14-day high-dose amoxicillin/proton pump inhibitor (PPI) dual therapies were 86.1% (3335/3875; 95% confidence interval (CI): 85.1–87.2%) by intention-to-treat (ITT) analysis and 87.3% (3232/3702; 95% CI: 86.2–88.4%) by per-protocol (PP) analysis. For 14-day high-dose amoxicillin/vonoprazan dual therapies, the rates were 87.4% (1085/1241; 95% CI: 85.5–89.2%) by ITT and 93.0% (1044/1124; 95% CI: 91.5–94.5%) by PP. In the penicillin-allergic population, 14-day tetracycline/vonoprazan dual therapy showed eradication rates of 92.0% (138/150) by ITT and 95.1% (135/142) by PP. In conclusion, 14-day tetracycline/vonoprazan dual therapy presents an effective option for eradicating *H. pylori* in patients allergic to penicillin. For those without a penicillin allergy, first-line treatments can include 14-day mono-antibiotic regimens, such as high-dose amoxicillin/PPI dual, high-dose amoxicillin/vonoprazan dual, and tetracycline/vonoprazan dual therapies.

## 1. Introduction

*Helicobacter pylori* (*H. pylori*) infection is the primary cause of chronic gastritis, gastric ulcer, duodenal ulcer, gastric adenocarcinoma, and gastric mucosa-associated lymphoid tissue lymphoma (MALToma) [1,2,3]. Recent guidelines recommend considering eradication therapy for all individuals with confirmed *H. pylori* infections [4,5]. In the treatment of *H. pylori* infection, antibiotic resistance significantly influences eradication outcomes. Recent data indicate that primary resistance to clarithromycin, metronidazole, and levofloxacin in *H. pylori* is on the rise [6,7]. Currently, the resistance rates for clarithromycin, amoxicillin, metronidazole, tetracycline, and levofloxacin are 56–71%, 0–8%, 35–74%, 0–4%, and 21–43%, respectively [7]. Due to increasing clarithromycin resistance, the effectiveness of standard triple therapy has diminished, with eradication rates now below 80% in most countries. Consequently, several strategies including bismuth quadruple therapy and non-bismuth quadruple therapy (such as sequential therapy, concomitant therapy, and hybrid therapy) have been proposed to enhance the eradication rate of first-line anti-*H. pylori* treatments [8,9,10]. While most international guidelines or consensuses favor bismuth quadruple therapy (BQT) as the first-line treatment in areas with high clarithromycin resistance [8,9,10], this approach presents challenges, such as its complex regimen and the relatively high occurrence of adverse events observed in extensive randomized studies [11,12,13,14]. In two large-scale randomized controlled trials [13,14], the frequencies of adverse events of BQT were 48% and 56%, respectively. The most frequently observed adverse events were taste distortion, darkened tongue, darkened stool, nausea, and abdominal discomfort [15,16,17,18,19].

Concomitant therapy is an effective regimen with proven success regardless of clarithromycin resistance in several studies [10]. It is a four-drug regimen containing proton pump inhibitor (PPI), clarithromycin, amoxicillin, and metronidazole, which are all administered for the entire duration of therapy. A meta-analysis demonstrated that concomitant therapy is more effective than standard triple therapy. Hybrid therapy is another non-bismuth quadruple therapy developed by our group [8]. It consists of a dual therapy with a PPI and amoxicillin for 7 days, followed by a quadruple regimen with a PPI, amoxicillin, clarithromycin, and metronidazole for 7 days. A pilot study of hybrid therapy showed that it achieved an eradication rate of 97.4% by intention-to-treat (ITT) analysis and 99.1% by per-protocol (PP) analysis in Taiwan [8]. A recent large multicenter randomized controlled trial documented that 14-day hybrid therapy and 14-day BQT had comparable efficacy in the treatment of *H. pylori* infection, and both could cure more than 90% of patients with *H. pylori* infections in areas of moderate clarithromycin resistance (17%) [14]. In addition, 14-day hybrid therapy had fewer adverse events than 14-day BQT. However, hybrid therapy requires an additional two antibiotics in the last 7 days, which can confuse patients and may dampen enthusiasm for its use in clinical practice. Reversing the sequence of drug administration (a quadruple regimen followed by a dual regimen) can simplify hybrid therapy; patients do not need to take additional medications during the course of treatment. A multicenter randomized trial demonstrated that 14-day reverse hybrid therapy had comparable eradication rate as 14-day BQT [13]. The novel therapy also had a lower frequency of adverse events than 14-day BQT. Nonetheless, all the bismuth quadruple, concomitant, hybrid, and reverse hybrid therapies require two to three antibiotics to eradicate *H. pylori*.

Currently, resistance rates to amoxicillin and tetracycline remain extremely low (<3%) in most countries [20,21]. An alternative dual therapy, combining a high-dose PPI with amoxicillin, maintains intragastric pH above 6.5, irrespective of the *CYP2C19* genotype, and ensures a constant plasma concentration of amoxicillin or tetracycline that exceeds the minimal inhibitory concentration (MIC) required to combat *H. pylori*. A randomized controlled trial in Taiwan demonstrated that the intention-to-treat eradication rate for mono-antibiotic therapy with high-dose amoxicillin and rabeprazole exceeded that of standard triple therapy (95% vs. 81%) [22]. Additional benefits include simplicity, fewer adverse events, and the avoidance of unnecessary antibiotics. However, growing evidence indicates that the effectiveness of high-dose amoxicillin/PPI dual therapy varies across countries.

Vonoprazan is a novel gastric acid suppression agent that functions as a potassium-competitive acid blocker (P-CAB) [23,24]. It exerts its acid-suppressive effect by reversibly inhibiting the H+/K+-ATPase enzyme [23,24]. Research has demonstrated that vonoprazan’s capacity to suppress gastric acid secretion exceeds that of proton pump inhibitors (PPIs). Additionally, it may enhance *H. pylori* therapy by optimizing gastric acid suppression and antimicrobial activity compared to PPIs. The primary metabolic pathway for vonoprazan involves cytochrome P450 3A4 (CYP3A4) in the liver, with additional metabolism by SULT2A1, CYP2C19, CYP2B6, and CYP2D6. Evidence, including several randomized controlled trials and non-randomized studies, has shown that a 7-day vonoprazan-based triple therapy is more effective than a 7-day PPI-based triple therapy as a first-line treatment for *H. pylori* infection [23,24]. Moreover, vonoprazan dual therapy has shown similar efficacy to vonoprazan triple therapy in clinical trials [25]. Recently, there has been growing interest in using a single sensitive antibiotic with potent acid suppression for *H. pylori* infection. These new mono-antibiotic regimens are simpler to administer; have fewer adverse events than standard triple, bismuth quadruple, and concomitant therapies; and may potentially become standard therapies for *H. pylori* infection in the future.

This article aims to assess the efficacy of 14-day mono-antibiotic therapies for the first-line treatment of *H. pylori* infection through recent randomized controlled trials and to develop a new algorithm for the treatment of *H. pylori* infection.

## 2. Article Search

This systematic review was conducted following the PRISMA (Preferred Reporting Items for Systematic Reviews and Meta-Analyses) guidelines to enhance the organization of its methodology. The central question addressed was, “In patients with *H. pylori* infection, can 14-day mono-antibiotic therapies achieve an eradication rate ≥ 90% according to per-protocol analysis in first-line treatment?” The bibliographic search was conducted using PubMed, Embase, and Cochrane Library electronic databases. The search terms included (“*Helicobacter pylori*” [MeSH] OR “*H. pylori*” [MeSH]) AND (“Therapy” [MeSH] OR “Treatment” [MeSH]), focusing on the publication’s abstract and/or title. The research was limited to clinical trials published in English from 1 October 2014 to 1 October 2024. Two investigators (C.A.S. and C.B.S.) independently carried out the search and study selection, with a third author (P.I.H.) assisting in cases of disagreement over article selection.

The inclusion criteria were (1) human studies on 14-day high-dose amoxicillin/PPI dual, 14-day high-dose amoxicillin/vonoprazan dual, and 14-day tetracycline/vonoprazan dual therapies for first-line *H. pylori* treatment; (2) participants over the age of 18; (3) randomized controlled trials; (4) articles reporting treatment efficacy; and (5) publications in English. The exclusion criteria included (1) treatments shorter than 14 days; (2) amoxicillin doses below 3 g/day; (3) unconfirmed *H. pylori* status pre- and post-treatment via at least one of the following tests: rapid urease test, histology, culture, or urea breath test; (4) assessment of *H. pylori* post-treatment status less than four weeks after treatment completion; (5) studies not reporting results for both ITT and PP analyses; and (6) studies published only in abstract form.

**Figure 1:**
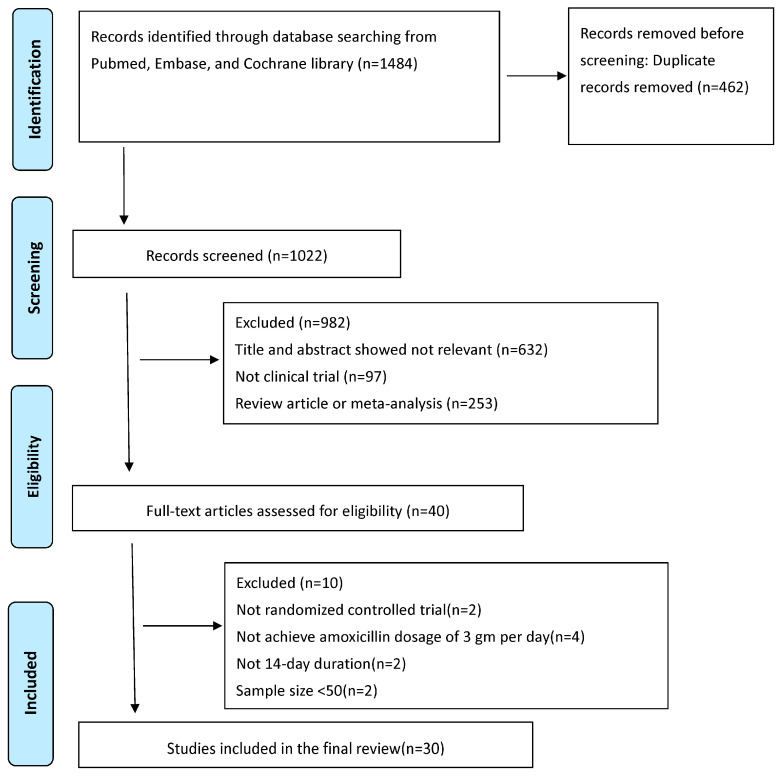
PRISMA diagram of the literature search.

All relevant data matching the study’s objectives were collected. Two authors (C.A.S. and C.B.S.) performed this task; in cases of disagreement, a third author (P.I.H.) was consulted. Specifically retrieved were the author’s name, year of publication, study country, sample size, and eradication rates by ITT and PP analyses. Initially, 1484 articles were found; 1444 were excluded during the initial screening due to irrelevance for being meta-analyses, review articles, or not clinical trials. After removing these articles, the remaining articles were screened by reading their titles and abstracts. Subsequently, one additional article was excluded due to duplication, leaving 40 for full-text evaluation. The previously defined eligibility criteria led to the final selection of 30 studies. For data analysis, the MedCalc v23.0.2 statistical software package (MedCalc Software Ltd., Ostend, Belgium) was used. Figure 1 presents the flow diagram of the literature search.

## 3. High-Dose Amoxicillin/PPI Dual Therapy

Table 1 summarizes the ITT and PP eradication rates of high-dose amoxicillin/PPI dual therapy for the first-line treatment of *H. pylori* infection in randomized control trials conducted between 2014 and 2024 [15,16,17,18,19,22,26,27,28,29,30,31,32,33,34,35,36,37,38]. The eradication rates from ITT analysis range from 65.7% to 95.3%, and from PP analysis, they range from 71.0% to 96.6%. The pooled eradication rates for 14-day high-dose amoxicillin/PPI dual therapies were 86.1% (3335/3875; 95% Confidence Interval (CI): 85.1–87.2%) by ITT analysis and 87.3% (3232/3702; 95% CI: 86.2–88.4%) by PP analysis. These data suggest that the eradication rates of high-dose amoxicillin/PPI vary across countries and do not consistently achieve an eradication rate of ≥ 90%. For instance, a regimen comprising 750 mg of amoxicillin four times daily and 40 mg of esomeprazole three times daily for 14 days achieved a PP eradication rate of 95.7% (110/115) in Taiwan [27], whereas the PP eradication rate for the same regimen in a study from China was only 71.0% (71/100) [15,16,17,18,19,22,26,27,28,29,30,31,32,33,34,35,36,37,38].

The variable efficacies of high-dose amoxicillin/PPI dual therapy for *H. pylori* treatment may be attributed to differences in the prevalence of amoxicillin-resistant strains, the potencies and dosages of PPIs, ethnic differences in PPI metabolism, patient drug adherence, body surface area (BSA), and the amount of acidic food intake. A large multicenter randomized controlled trial conducted by our group indicated that amoxicillin-resistant strains and poor drug adherence were independent risk factors predicting eradication failure in high-dose amoxicillin/rabeprazole dual therapy, with odds ratios of 8.2 and 8.6, respectively [19]. In this study, the eradication rate for patients receiving high-dose amoxicillin/rabeprazole dual therapy was significantly lower in those with amoxicillin-resistant strains compared to those with susceptible strains (50% vs. 88%). Similarly, patients with poor drug adherence had a lower eradication rate than those with good adherence (40% vs. 87%). Another clinical study from China found that a BSA ≥1.69 m^2^ was the only independent predictor of eradication failure for high-dose amoxicillin/PPI dual therapy [39].

The pooled incidence of adverse events in these studies of high-dose amoxicillin/PPI dual therapy was 13.0% (484 out of 3804). The most frequently observed adverse events were diarrhea, abdominal distension, and nausea, which were generally mild.

**Table 1:** Eradication rates of 14-day high-dose amoxicillin/PPI dual, high-dose amoxicillin/vonoprazan dual, and tetracycline/vonoprazan dual therapies for the first-line treatment of *H. pylori* infection in randomized controlled trials from 2014 to 2024.

Author (Year)	Country	No. of Cases	Regimen	Test to Conform Eradication	Eradication Rate	Adverse Events
					ITT PP	
**14-day high-dose amoxicillin/PPI dual therapy**						
Yang et al. [22] (2015)	Taiwan	150	rabeprazole 20 mg qid, amoxicillin 750 mg qid	^13^C-UBT	95.3% (143/150) 96.6% (143/148)	23.0% (34/148)
Hu et al. [26] (2017)	China	87	rabeprazole 20 mg qid, amoxicillin 750 mg qid	^13^C-UBT	81.6% (71/87) 83.5% (71/85)	3.4% (3/87)
Tai et al. [27] (2019)	Taiwan	120	esomeprazole 40 mg tid, amoxicillin 750 mg qid	^13^C-UBT	91.7% (110/120) 95.7% (110/115)	9.6% (11/115)
Yang et al. [15] (2019)	China	116	esomeprazole 20 mg qid, amoxicillin 750 mg qid	^13^C-UBT	87.9% (102/116) 91.1% (102/112)	6.3% (7/112)
Yu et al. [28] (2019)	China	80	esomeprazole 40 mg bid, amoxicillin 1 gm tid	^13^C-UBT	92.5% (74/80) 96.1% (73/76)	7.5% (6/80)
Song et al. [29] (2020)	China	380	esomeprazole 20 mg qid, amoxicillin 750 mg qid	^13^C-UBT	87.1% (331/380) 92.4% (329/356)	17.6% (66/375)
Hwong-Ruey et al. [30] (2020)	Malaysia	97	rabeprazole 20 mg qid, amoxicillin 1 gm qid	^13^C-UBT	92.8% (90/97) 93.8% (90/96)	20.5% (20/97)
Shen et al. [31] (2022)	China	496	esomeprazole 20 mg qid, amoxicillin 750 mg qid	^13^C/^14^C-UBT	88.31% (438/496) 91.63% (438/478)	13.3% (66/496)
Guan et al. [16] (2022)	China	350	esomeprazole 20 mg qid, amoxicillin 1 gm tid	^13^C/^14^C-UBT	89.4% (313/350) 90.6% (308/340)	12.9% (45/349)
Han et al. [32] (2022)	China	315	esomeprazole 20 mg qid, amoxicillin 750 mg qid	^13^C/^14^C-UBT	88.6% (279/315) 90.4% (274/303)	13.7% (43/314)
Shao et al. [17] (2022)	China	120	rabeprazole 20 mg tid, amoxicillin 1 gm tid	^13^C-UBT	85.8% (103/120) 89.6% (103/115)	13.0% (15/115)
Bi et al. [33] (2022)	China	329	esomeprazole 40 mg tid, amoxicillin 1 gm tid	^13^C/^14^C-UBT	75.4% (248/329) 81.3% (248/305)	11.1% (34/305)
Liu et al. [18] (2023)	China	422	esomeprazole 20 mg qid, amoxicillin 1 gm tid	^13^C-UBT	90.3% (381/422) 93.6% (381/407)	13.5% (55/407)
Hsu et al. [19] (2023)	Taiwan	306	rabeprazole 20 mg qid, amoxicillin 750 mg qid	^13^C-UBT	83% (255/306) 87% (253/291)	13.0% (40/305)
Ding et al. [34] (2023)	China	134	esomeprazole 40 mg bid, amoxicillin 1 gm tid	^13^C-UBT	73.1% (98/134) 83.1% (98/118)	6.0% (8/134)
Yun et al. [35] (2023)	China	108	esomeprazole 40 mg tid, amoxicillin 750 mg qid	^13^C-UBT	65.7% (71/108) 71.0% (71/100)	2.0% (2/100)
Zhang et al. [36] (2023)	China	101	ilaprazole 5 mg bid, amoxicillin 1 gm tid	^13^C-UBT	92.1% (93/101) 94.9% (93/98)	13.9% (14/101)
Macedo et al. [37] (2023)	Portugal	50	esomeprazole 40 mg bid, amoxicillin 1000 mg alternating with amoxicillin 500 mg qid	SAT	96.2% (48/50) 95.9% (47/49)	2.0% (1/50)
Valizadeh et al. [38] (2024)	Iran	114	esomeprazole 40 mg bid, amoxicillin 1 gm tid	SAT	76.3% (87/114) 79.1% (87/110)	12.2% (14/114)
All					86.1% (3335/3875) 87.3% (3232/3702)	13.0% (484/3804)
**14-day high-dose amoxicillin/vonoprazan dual therapy**						
Chey et al. [40] (2022)	USA	324	vonoprazan 20 mg bid, amoxicillin 1000 mg tid	^13^C-UBT	78.5% (208/265) 81.2% (177/218)	29.9% (104/348)
Yang et al. [41] (2023)	China	200	vonoprazan 20 mg bid, amoxicillin 1000 mg tid	^13^C/^14^C-UBT	86% (172/200) 92.5% (172/186)	9.5% (17/200)
Peng et al. [42] (2023)	China	158	vonoprazan 20 mg bid, amoxicillin 750 mg qid	^13^C-UBT	89.9% (142/158) 97.9% (142/145)	19.0% (30/158)
Hu et al. [43] (2023)	China	97	vonoprazan 20 mg bid, amoxicillin 1000 mg tid	^13^C-UBT	88.6% (86/97) 95.5% (86/90)	16.67% (15/90)
Hu et al. [44] (2023)	China	55	vonoprazan 20 mg bid, amoxicillin 1000 mg tid	^13^C-UBT	87.3% (48/55) 95.9% (47/49)	20.0% (11/55)
Jiang et al. [45] (2024)	China	200	vonoprazan 20 mg bid, amoxicillin 1000 mg tid	^14^C-UBT	94.0% (188/200) 97.9% (188/192)	19.0% (38/200)
Huang et al. [46] (2024)	China	102	vonoprazan 20 mg bid, amoxicillin 1000 mg tid	^13^C^/14^C-UBT	92.2% (94/102) 93.9% (93/99)	13.7% (14/102)
Cheung et al. [47] (2024)	China	100	vonoprazan 20 mg bid, amoxicillin 1000 mg tid	^13^C-UBT	96.0% (96/100) 96.7% (89/92)	39.0% (39/100)
Liu et al. [48] (2024)	China	64	vonoprazan 20 mg bid, amoxicillin 1000 mg tid	^13^C^/14^C-UBT	79.7% (51/64) 94.3% (50/53)	7.8% (5/64)
All					87.4% (1085/1241) 93.0% (1044/1124)	20.7% (273/1317)
**14-day tetracycline/vonoprazan dual therapy**						
Gao et al. [49] (2024)	China	150	vonoprazan 20 mg bid, tetracycline 500 mg tid	^13^C-UBT	92.0% (138/150) 95.1% (135/142)	14.0% (21/150)

Abbreviations: UBT, urea breath test; SAT, stool antigen test; bid, twice a day; tid, three times a day; qid, four times a day.

## 4. High-Dose Amoxicillin/Vonoprazan Dual Therapy

Table 1 lists the ITT and PP eradication rates of high-dose amoxicillin/vonoprazan dual therapy for the first-line treatment of *H. pylori* infection in randomized control trials from 2014 to 2024 [40,41,42,43,44,45,46,47,48]. The ITT analysis shows eradication rates ranging from 78.5% to 96.0%, and PP analysis shows rates from 81.2% to 97.9%. The pooled eradication rates for 14-day high-dose amoxicillin/vonoprazan dual therapies were 87.4% (1085/1241; 95% CI: 85.5–89.2%) by ITT and 93.0% (1044/1124; 95% CI: 91.5–94.5%) by PP. The efficacy of high-dose amoxicillin/vonoprazan dual therapy also varies across countries and does not consistently achieve an eradication rate of ≥ 90%. In China, nearly all randomized controlled trials reported PP eradication rates of ≥ 90% for this therapy. However, in the USA and Taiwan, PP eradication rates were only 81.2% [40] and 87.2% [50], respectively. Thus, optimizing high-dose amoxicillin/vonoprazan dual therapy is essential before it can be established as a standard first-line treatment for *H. pylori*.

Currently, the risk factors predicting eradication failure of 14-day high-dose amoxicillin/vonoprazan dual therapy remain unclear. In a randomized controlled trial from Taiwan [50], poor drug adherence was identified as an independent risk factor predicting eradication failure, with an odds ratio of 8.4. Patients with poor drug adherence had a significantly lower eradication rate compared to those with good adherence (60.0% vs. 87.7%). A clinical study from Japan showed that a high BSA was a risk factor for eradication failure with 7-day regular-dose vonoprazan dual therapy (vonoprazan twice daily plus amoxicillin 750 mg twice daily) [51]. Failure of high-dose vonoprazan dual therapy for *H. pylori* eradication was associated with higher BSA (eradication rate: 79.6% in those with BSA ≥1.723 vs. 90.8% in patients with BSA <1.723).

The pooled incidence of adverse events in these studies of high-dose amoxicillin/vonoprazan dual therapy was 20.7% (273 out of 1317). No serious adverse reactions were reported with high-dose amoxicillin/vonoprazan dual therapy. The main adverse events included nausea, diarrhea, and abdominal distension. Among them, two important studies by Chey et al. [40] and Cheung et al. [47] reported adverse event incidences of 29.9% and 39%, respectively. The most common adverse events identified in the analysis of the two studies were diarrhea, with rates of 5.2% and 12.0%, respectively.

Bismuth salts enhance the effectiveness of antibiotics, yielding synergistic effects that boost eradication efficacy in anti-*H. pylori* therapy. Evidence shows that bismuth can inactivate bacterial urease, respiratory enzymes (such as F1-ATPase), and alcohol dehydrogenase. Additionally, bismuth drugs disrupted several essential pathways of pathogens, including oxidative defense systems and bacterial pH-buffering ability, and bacterium–host cell adhesion. Adding bismuth to the standard 14-day triple therapy can improve cure rates [52]. Specifically, the major effect of bismuth is to increase the cure rate by an additional 30–40% in the treatment of resistant strains [52]. A single randomized controlled trial by Liang et al. has reported on the efficacy of amoxicillin/vonoprazan/bismuth triple therapy [53]. This regimen, consisting of vonoprazan 20 mg twice daily, amoxicillin 750 mg three times daily, and bismuth 220 mg twice daily for 14 days, achieved an ITT eradication rate of 83.7% and a PP eradication rate of 90.9%. The study found no differences in eradication efficacy between this 14-day triple therapy and a quadruple regimen that includes esomeprazole, bismuth, clarithromycin, and amoxicillin for *H. pylori* eradication [53]. Recently, we conducted a pilot study to assess the efficacy and safety of high-dose amoxicillin/vonoprazan/bismuth triple therapy. The regimen included tripotassium dicitrato bismuthate (300 mg four times daily), high-dose amoxicillin (750 mg four times daily), and vonoprazan (20 mg twice daily) for 14 days. The eradication rates of this novel therapy as a first-line treatment were 95.6% (43/45) by ITT analysis and 100.0% (43/43) by PP analysis [54]. Further research is warranted to determine whether high-dose amoxicillin/vonoprazan/bismuth triple therapy can achieve higher eradication rates than the regular-dose regimen.

## 5. Tetracyline/Vonoprazan Dual Therapy

Dual therapy using high-dose PPI or standard-dose vonoprazan plus high-dose amoxicillin has been proven effective and safe for treating *H. pylori* in Asia [55,56]. However, this amoxicillin-based dual therapy may not be suitable for individuals allergic to penicillin or those infected with amoxicillin-resistant *H. pylori* strains. Tetracycline, one of the most effective antibacterial drugs against *H. pylori*, has a resistance rate generally between 1.2% and 3.3% [57]. Table 1 presents the ITT and PP eradication rates of 14-day tetracycline/vonoprazan dual therapy for the first-line treatment of *H. pylori* infection in randomized controlled trials conducted between 2014 and 2024 [49]. There was only one randomized controlled trial that assessed this novel regimen for *H. pylori* eradication. In this study, Gao et al. [49] evaluated the efficacy of vonoprazan/tetracycline dual therapy, which included vonoprazan 20 mg twice daily and tetracycline 500 mg three times daily for 14 days, in patients with a history of penicillin allergy. This regimen achieved an ITT eradication rate of 92.0% and a PP eradication rate of 95.1%. The study found no significant differences in eradication rates between the 14-day tetracycline/vonoprazan dual therapy and standard bismuth quadruple therapy, although the former resulted in fewer adverse events. Further research is essential to explore the efficacy of 14-day tetracycline/vonoprazan dual therapy in patients without a history of penicillin allergy.

## 6. Advantages of High-Dose Dual Therapy Versus Standard Triple and Bismuth Quadruple Therapies

Dual therapies combining an antibiotic with a PPI or potassium competitive acid blocker (PCAB) for *H. pylori* infection offer simplicity, minimal adverse effects, and the reduction of unnecessary antibiotic use. Dual therapies were associated with fewer adverse events, primarily including nausea, diarrhea, and abdominal discomfort. However, these were mild and did not significantly disrupt the patients’ daily activities. An initial trial in Taiwan of high-dose rabeprazole/amoxicillin dual therapy showed that the intention-to-treat eradication rate was superior to that of standard triple therapy (95% vs. 81%) [22]. However, current reviews indicate that eradication rates for high-dose amoxicillin/PPI therapy vary internationally and do not consistently exceed 90%. Eradication rates by PP analysis range from 71.0% to 96.6% in randomized controlled trials (Table 1). A recent large-scale trial demonstrated that bismuth quadruple therapy achieved higher eradication rates than high-dose dual therapy (PP analysis: 95% vs. 87%), albeit with more adverse events [19]. Vonoprazan, a potent acid inhibitor, has shown superior acid suppression compared to PPIs. A significant study from the United States and Europe found that 14-day vonoprazan triple therapy was more effective than 14-day PPI triple therapy [40]. No significant differences in eradication rates were observed between 14-day high-dose amoxicillin/vonoprazan dual therapy and 14-day amoxicillin/clarithromycin/vonoprazan triple therapy. Moreover, a recent trial revealed that 10-day high-dose vonoprazan–amoxicillin dual therapy in China achieved similar eradication rates to bismuth quadruple therapy, but with fewer adverse events, as a first-line treatment [58]. However, the efficacy of high-dose amoxicillin/vonoprazan dual therapy still varies by country, with PP analysis showing eradication rates between 81% and 98% (Table 1). Only one randomized controlled trial has evaluated vonoprazan/tetracycline dual therapy [49], finding no significant differences in eradication rates compared to standard bismuth quadruple therapy, but with fewer adverse events.

The bactericidal effect of amoxicillin against *H. pylori* is both time- and pH-dependent, as amoxicillin is more stable at higher intragastric pH levels. Dual therapies involving a PPI and amoxicillin administered twice daily have not achieved satisfactory outcomes [59]. Instead, their effectiveness can be enhanced by administering both drugs at higher doses and frequencies [60]. It has been observed that eradication rates are generally higher with four-times-daily dosing compared to three times daily, as maintaining a steady plasma concentration of amoxicillin above the minimum inhibitory concentration is crucial for its bactericidal effect against *H. pylori* [61]. This is because it is critical to maintain a steady plasma concentration of amoxicillin above the MIC with more frequent dosing for its bactericidal effect against *H. pylori*.

This systematic review has several limitations. First, the majority of the included studies originate from China, potentially limiting the applicability of the findings in other regions. Second, the number of included articles is small, particularly those addressing tetracycline/vonoprazan dual therapy. Third, only two studies are from Western countries (USA and Portugal). Therefore, the applicability of the therapies recommended in this review should be further validated in Western contexts.

## 7. Conclusions

**Figure 2:**
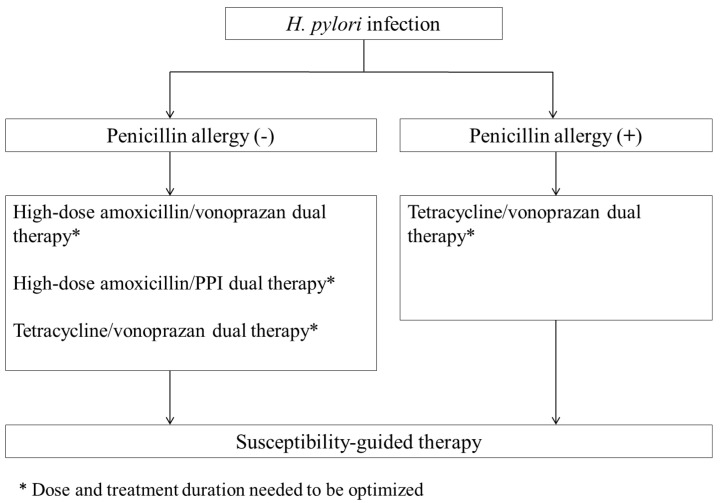
Algorithm for mono-antibiotic anti-H. pylori therapy of Helicobacter pylori infection.

Although most international consensuses and guidelines recommend anti-*H. pylori* therapy based on local rates of clarithromycin resistance, there is growing interest in using a single sensitive antibiotic in combination with potent acid suppression for *H. pylori* infection. This systemic review shows that 14-day dual therapies combining an antibiotic with a PPI or vonoprazan can achieve a high eradication rate for *H. pylori* infection and have fewer adverse effects than bismuth quadruple therapy. These encouraging results suggest the potential for developing simple and effective mono-antibiotic regimens for *H. pylori* treatment. In Figure 2, we propose a new algorithm of mono-antibiotic therapies for *H. pylori* infection based on the history of penicillin allergy. In patients with a penicillin allergy, 14-day tetracycline/vonoprazan dual therapy is a viable option for eradicating *H. pylori*. For those without a penicillin allergy, first-line treatments such as 14-day high-dose amoxicillin/PPI dual, high-dose amoxicillin/vonoprazan dual, and tetracycline/vonoprazan dual therapies are potentially effective mono-antibiotic options for *H. pylori* eradication. However, current dual therapies based on amoxicillin or tetracycline require taking antibiotics three to four times daily. A significant drawback of this regimen is poor adherence to treatment. Therefore, optimizing the dosage, administration, and duration of these novel dual therapies is essential before they can be established as a standard first-line treatment.

## Data Availability

No new data were created or analyzed in this study.
